# A teaching proposal for a short course on biomedical data science

**DOI:** 10.1371/journal.pcbi.1012946

**Published:** 2025-04-14

**Authors:** Davide Chicco, Vasco Coelho

**Affiliations:** 1 Dipartimento di Informatica Sistemistica e Comunicazione, Università di Milano-Bicocca, Milan, Italy; 2 Institute of Health Policy Management and Evaluation, University of Toronto, Toronto, Ontario, Canada; Retired, Montreal, Quebec, CANADA

## Abstract

As the availability of big biomedical data advances, there is a growing need of university students trained professionally on analyzing these data and correctly interpreting their results. We propose here a study plan for a master’s degree course on biomedical data science, by describing our experience during the last academic year. In our university course, we explained how to find an open biomedical dataset, how to correctly clean it and how to prepare it for a computational statistics or machine learning phase. By doing so, we introduce common health data science terms and explained how to avoid common mistakes in the process. Moreover, we clarified how to perform an exploratory data analysis (EDA) and how to reasonably interpret its results. We also described how to properly execute a supervised or unsupervised machine learning analysis, and now to understand and interpret its outcomes. Eventually, we explained how to validate the findings obtained. We illustrated all these steps in the context of open science principles, by suggesting to the students to use only open source programming languages (R or Python in particular), open biomedical data (if available), and open access scientific articles (if possible). We believe our teaching proposal can be useful and of interest for anyone wanting to start to prepare a course on biomedical data science.

## Introduction

During the second semester of the last academic year, we taught a biomedical data science course within the Master Degree program on Data Science of our Università di Milano-Bicocca in Milan (Italy, EU). This situation gave us the possibility to prepare up-to-date, modern content for our lectures, and to take stock of the situation on the steps to perform a biomedical data science analysis correctly and precisely.

The key principle of our teachings is to avoid the automated, blind usage of machine learning, computational statistics, and data science programs and tools, and to evaluate each step and its results critically each time.

Moreover, during our classes we highlighted the importance of preprocessing steps to apply on biomedical data. What we saw many times in the scientific literature was the wrong, blind application of machine learning methods to biomedical datasets, without data cleaning or data preparation steps. We tried to curb this problem by teaching the importance of these preprocessing phases to the students attending our classes.

**Fig 1:**
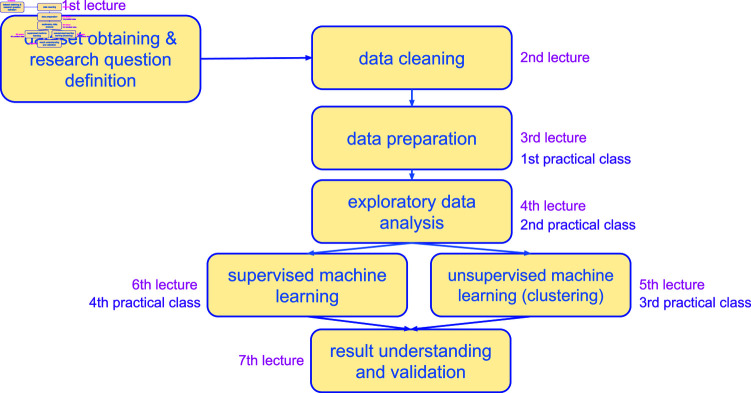
A schematic representation of our short course. We broadly covered each topic in one corresponding lecture.

We list the theoretical lectures of our classes in Fig. 1. hese lectures were given in person, in a classroom within our university campus. Moreover, we also gave some practical computer classes where students had the chances to apply the concepts studied through R scripts and public on data of electronic health records (EHRs).

Our short course was attended by approximately ten students, all of whom held a Bachelor’s degree in computer science, computer engineering, mathematics, physics, or statistics. This cohort consisted entirely of male students, approximately 22 years old, who had no specific knowledge of biology or medicine. We cannot know the nationality of the students, but we believe they were probably all Italians. The lectures of our short course and all the Data Science master degree, however, were and are taught in English.

We describe the content of our course here in this manuscript so that it can be useful for anyone who has the opportunity to prepare a biomedical data science teaching unit.

## Course content

**1st lecture: Dataset obtaining and research question definition.** In the first class (Fig. 1), we described the most common data types for biomedical data: medical images, electronic health records, next generation sequencing (such as microarray gene expression, bulk RNA-seq, single-cell RNA-seq, ATAC-seq), and physiologic data (such as electrocardiography and electrocardiography). We highlighted the fact that all these data are recorded for clinical purposes, and not for scientific research goals; in this context, we also recommended that the students to consider and to document all the patients inclusion and exclusion criteria for a specific dataset.

We then explored the two main scenarios on how a researcher can obtain a biomedical dataset: by receiving it directly from a medical doctor, or by finding it online as a public resource. Open biomedical datasets, in fact, can be found on public repositories and through dataset search engines, such as Google Dataset Search, re3data.org, PhysioNet, Zenodo, Kaggle, University of California Irvine Machine Learning Repository, Figshare, UK Biobank, or dbGaP. Open datasets, moreover, can be uncovered also in the supplementary material of scientific journals, such as PLOS One for example [1,2].

**Fig 2:**
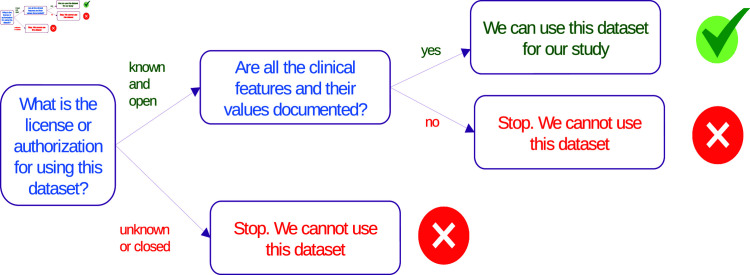
A schematic representation of the checks to do before using a dataset. During our course, we reaffirmed the necessity of discarding datasets for which there is no sufficient documentation or no license of usage.

In both cases, we explained to our students that checking the data privacy and protection license of a dataset before analyzing it is of fundamental importance. We recommended not to use the data blindly. If no license, no written authorization to use the data, and no information about the privacy is present, we advice to discard the dataset and look for another one (Fig. 2). Without these important pieces of information, the project on that dataset must stop there. Period.

On the contrary, if the proper authorizations to use the dataset are present (such as a Creative Commons CC BY 4.0 Deed Attribution 4.0 International license), you can proceed with the data science project. Users need to understand they are not authorized to try to re-identify the names of the patients of the dataset.

Another relevant check to do before deciding if a dataset can be analyzed or not is about documentation: you can employ a dataset for your scientific analysis only if all the features and their values of the dataset are documented (Fig. 2). Absence of documentation for datasets can generate cascades of problems in computational research [3].

Of course, this first step on obtaining a dataset is not intended to be automated, and should be done manually by a student or a researcher at the beginning of a biomedical data science project.

Together with obtaining the dataset, another key aspect of beginning a data science project is having a proper biomedical question to investigate. It is difficult to determine whether the egg or the chicken came first: should you first have a sound biomedical research question and then look for a dataset, or should you first find an available dataset and then design the proper research question? In our educational context, since it is rare for students to have a well-defined biomedical question to investigate, we approach the situation by first obtaining a dataset and then formulating a research question.

**Fig 3:**
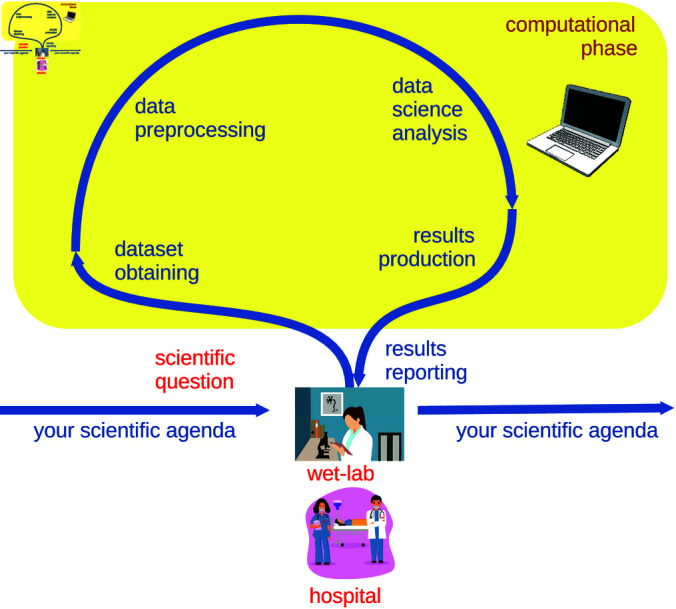
Schematic representation of a sound biomedical research project cycle. A sound biomedical question originates in a hospital from medical doctors or in a wet lab from biologists who identify a current gap or problem in biomedical research, clinical practice, or understanding of biology. A scientific question formulated by biomedical engineers or health informatics researchers, without the input of biomedical scientists, might be poorly posed or misleading. Biomedical data scientists take custody of the research question from the hospital medical doctors or wet lab biologists, study the dataset, and preprocess it for computational analysis. They use data science methods to infer new knowledge from these data and eventually deliver their scientific results back to the clinical doctors or biologists where the scientific question originated. The medical doctors or biologists review the results, provide feedback, comments, prompts, and insights, and may adjust their strategies for treatments and therapies for patients or enhance their understanding of human biology. This image is an adaptation of Figure 4 of [4], published under the Creative Commons CC-4.0 Deed license. The medical doctors illustration was released under the Creative Commons CC-4.0 Deed license on IconScout.com. The wet-lab illustration was released under the Creative Commons CC0 1.0 Universal license on StockVault.net. The laptop illustration was released under the Creative Commons Attribution 4.0 International license on Wikimedia.org.

Defining a clear, sound, and valid biomedical research question is one of the most important steps in a scientific study, and this phase should be done in synergy with a medical doctor or a wet-lab biologist [4,Tip 1] (Fig. 3). Medical doctors and biologists, of course, ideally should be involved in all the phases of the study, if possible, in an iterative way: one of them should guide the data scientist not only during the research question definition, but also during preprocessing, exploratory data analysis, and results understanding and validation. In reality, however, things work quite differently: physicians and wet-lab researchers are so busy that, more realistically, one can expect to meet them only at the beginning of the study (for the research question definition) and at the end (for the results assessment).

A research question addresses a real issue in biomedical research that the data science analysis on the selected dataset can attempt to answer [5]. A good way to test and evaluate a question is to attempt to answer the questions posed by the *Heilmeier Catechism* [6,7]. To generate a realistic and well-grounded research question, one needs to be familiar with the current state of the art on the investigated theme in the scientific literature. Therefore, we taught students that browsing the most recent biomedical literature related to the topic is pivotal at the beginning of a data science study, by leveraging online literature search engines (Google Scholar, Scopus, DBLP, PubMed, IEEE *Xplore*, etc).

To recap: get the dataset; define a reasonable, innovative biomedical research question with a wet-lab biologist or a medical doctor; study the recent literature on that topic; update the research question if necessary; check if the dataset can solve that biomedical question; and finally double-check your biomedical question again (Fig. 3).

**2nd lecture: Data cleaning.** Once it is confirmed that a biomedical dataset can be analyzed for a scientific project, the first step to carry on is data preprocessing, a phase that we taught in the second and third lectures of our course (Fig. 1). The goal of biomedical data cleaning is to ensure that the data used in a data science analysis are accurate, consistent, and reliable. This phase is crucial in the biomedical sciences because the quality of the data directly can impact the validity of the research findings and subsequent consequences [3].

In our course and in this article, we divide data preprocessing into two different phases:

- Data cleaning includes steps that affect small, limited portions of the dataset;
- Data preparation includes steps that affect a big portion of the dataset;
- Data preprocessing: data cleaning and data preparation.

These three terms (data cleaning, data preparation, and data preprocessing) are used interchangeably in the scientific literature.

In the first step of the pipeline, that we call *data cleaning*, we taught how to spot duplicates, errors, inconsistencies, and outliers. By *duplicates*, we indicate two features (columns) having identical values; by *errors* non-sense values (for example, age equal to –10); by *inconsistencies*, we indicate pairs of values that make sense alone but do not make sense together (for example, sex == male && ovarian_cancer_diagnosis == TRUE).

Identifying duplicates is straightforward: you need to compare all the possible pairs of features of a dataset and spot the identical ones, which is a quite easy operation in R or Python. Errors can be easy to notice for well-known variables such as age, but can be hard to spot for biomedical specific variable names. For this goal, we suggest to compare the ranges of each feature with the content of the documentation.

Outliers are exceptional points of the dataset, whose values are outside the mean of a feature. As a rule of thumb, we suggest to label as *outliers* all the points that are at least five times higher or smaller than the average value of a variable. Be careful: outliers are not always incorrect, they can be correct too. For example, a data point saying sex == male && breast_cancer_diagnosis == TRUE might seem wrong but can be correct: breast cancer, in fact, can affect men as well. Statistics say that 10% of patients with breast cancer are men [8], and therefore that data entry could be a proper, correct outlier.

In other cases, of course, outlier can represent just wrong data. If the dataset consists of patients diagnosed with diabetes type 1, which is the children diabetes kind, and one of the patients has 90 years as age recorded at first diagnosis, there must be a mistake.

Regarding errors, duplicates, inconsistencies, and wrong outliers, data scientists have two options: removing them completely or replacing them, using the same techniques that can be employed for missing data replacement [9,Tip 4]. Removing these data points is an easy step and can be done with low data loss in big dataset. A loss of information would happen in this case. Replacing these data instances with artificially created, realistic, alternative data points deduced statistically from the rest of the dataset would not affect the data size, but would introduce synthetic data within the dataset. In this case, the dataset could no longer be considered fully pure. We therefore suggest using synthetic data only if it constitutes a small proportion of the dataset (at most 10%) to avoid unrealistic outcomes and, in any case, to document this step thoroughly.

**3rd lecture: Data preparation.** In the following class about data preparation, we presented three main concepts: data transformation, missing data detection and handling, and data unbalance detection and handling. The goal of biomedical data preparation is to make a dataset ready and suitable for computational analyses, ensuring that subsequent outcomes and results can be interpreted without ambiguity or doubt.

Data transformations include simple changes to variables names or the generation of new variables inferred from the existing features. Name changes are not necessary, but suggested when variables having misleading or wrong names. A typical example, that we saw multiple times, is the usage of the name *gender* when the real meaning is *sex*. In these cases, it is also a good practice to include the meaning of the variable content in the variable name. For example, if 0 means men and 1 means women, a good name for that feature would be sex_0male_1female.

On the other hand, data transformations are needed to encode non-binary data in the correct way. That is the case of features having string values. Strings that have a numerical value can easily be mapped into ordinal values that preserve the mathematical meaning (for example, the values of the age feature *kid*, *teenager*, *adult*, and *elderly* can be mapped into a new variable having values 1, 2, 3, and 4, respectively).

However, variables not having a numerical value must not be mapped into numbers. If a feature indicated the site of a tumor and had possible values *breast*, *lung*, *kidney*, and *prostate*, it would be clearly a mistake to map these strings into the 1, 2, 3, and 4 numbers, because they have no mathematical meaning. In these cases, we recommend the usage of algorithms such as one-hot encoding [10], a simple technique that transform the value of the string variable into a new Boolean feature, representing the same information. In the previous example of the sites of a tumor, the new introduced variables would be breast_binary, lung_binary, kidney_binary, and prostate_binary: each of them would have value 1 if a patient had a tumor in that specific site, or 0 otherwise. The original site of tumor variable would then be eliminated before the scientific analysis.

Missing data are another issue that can be found in some datasets: some data entries can be partial because they were not recorded for all the patients, or because the patient did not take some medical exams in some periods [11,12].

Several techniques exist to handle missing data [9,Tip 4], whose realistic replacements can be inferred from the rest of the dataset [13,14]. Another option is to completely eliminate the features or the patients’ profiles which have missing data, but this approach would cause a loss of information, as mentioned earlier.

For binary classification tasks, in the data cleaning lecture we also described data imbalance handling, which happens when one of the two classes (zeros or ones) is overly more represented in the dataset than the other one. A common operation here is to create artificially new data instances of the minority class to the dataset, or to remove some data instances of the majority class from the dataset. Several techniques for handling data imbalance exist, such as SMOTE (synthetic minority over-sampling technique) [15] for example. During the lecture, we reaffirmed the importance of applying data imbalance handling techniques *only* on the training set, and not on the test set, to avoid corrupting the machine learning pipeline through *data snooping* [16,17].

**1st practical class: Data preprocessing.** During the first practical lecture we taught the data cleaning and preparation steps seen during the first three lectures, on a dataset [18] containing EHRs of patients with type 1 diabetes. Initially, we checked the dataset license and we found out that it is distributed under the terms of the Creative Commons Attribution License (CC BY 4.0), which permits unrestricted use, distribution, and reproduction in any medium, provided the original author and source are credited. As a second step, we collected some information about the disease, both from the introduction of the original paper [18], and online from the website of the World Health Organization (WHO) [19]. After getting an idea about the disease, we downloaded the CSV file of the dataset from Figshare indicated in the *supporting information* section of [18].

To have a look at the dataset, we loaded it into the memory of our computers using the R programming language and the RStudio integrated development environment (IDE) [20]. For reproducibility purposes we set a seed and we installed the pacman [21] library for an easier installation and loading of our package dependencies. We installed the dplyr [22], ggplot2 [23] and pastecs [24] R packages. We executed the standard dim(), summary(), and str() R commands on the dataset, and checked if there were duplicate features that should be eliminated. The features *TDD, basal* and *bolus* were reported as both absolute values and per kilogram ratios: we decided to keep the absolute values and drop the per kilogram ratios, deriving the new *weight_kg* feature as the product between the *TDD* feature and the inverse of the *TDD/weight_kg* features, doing so we reduced the total number of features without losing any information about the records. As a further data cleaning step, we detected errors among the *age, duration_of_diabetes* and *BMI* features, given their ranges in Table 2 of [25]: we decided to drop records with feature values outside the reported ranges. Additionally, we detected a few inconsistencies between the *age* and the *duration_of_diabetes* features: we dropped the records containing the former lower than the latter.

Finally, we applied the data preparation steps: data transformation of feature names and categories, and missing and unbalanced data handling methods. The *gender* and *insulin_regimen* features were converted from binary categories into the numerical values of 0 and 1. For clarification purposes, we also improved the naming of the *gender* feature into *sex_0man_1woman*, *insulin_regimen_binary* into *insulin_regimen_0CSII_1MDI*, *BMI* into *body_mass_index*, *age* into *age_years*, *duration_of_diabetes* into *diabetes_duration_years*, *OC* into *total_osteocalcin*, *SMI* into *skeletal_muscle_mass_index*, *TDD* into *total_daily_dose_of_insulin*, and *ucOC* into *undercarboxilated_osteocalcin*. We leveraged the Multivariate Imputation by Chained Equations (MICE) method package [13] to impute missing data. The dataset was split into training and test sets according to a 80%-20% guideline [26], by randomly selecting patients. We set the *insulin_regimen_binary* feature as the target variable, so that we could elaborate a classification of the patients based on this insulin condition from other variables,and we computed the percentage of the majority class over the minority class, and oversampled the minority class by generating new synthetic records using the synthetic minority oversampling technique (SMOTE) [27] method.

**4th lecture: Exploratory data analysis (EDA).** Once the dataset is preprocessed and ready to be used, most of researchers and students would immediately apply machine learning methods, trying to infer something about the dataset through computational intelligence methods. We disagree on this approach: before applying computational intelligence to infer new trends among the data, we believe an exploratory data analysis (EDA) based on simple statistics, visualization, and dimensionality reduction should be carried out [28]. That is what we teach to the students of our course.

John Tukey defined exploratory data analysis steps this way:

“Procedures for analyzing data, techniques for interpreting the results of such procedures, ways of planning the gathering of data to make its analysis easier, more precise or more accurate, and all the machinery and results of (mathematical) statistics which apply to analyzing data”. [29]

In our lesson, we divided the exploratory data analysis into three parts: quantitative description, statistical correlations, dimensionality reduction and its visualization. Regarding quantitative descriptions we recommend to compute the descriptive statistics of the dataset, by deducing the number of rows (usually it is number of patients or observations), the number of columns, the sparsity of the dataset (percentage of zeros), and the number and percentage of missing data in total. For each feature (column), we advice to calculate mean, median, standard deviation, minimum, and maximum, number of missing values (NAs) and its percentage. For each variable, we recommend to analyze the distribution of its values by plotting histograms.

**Fig 4:**
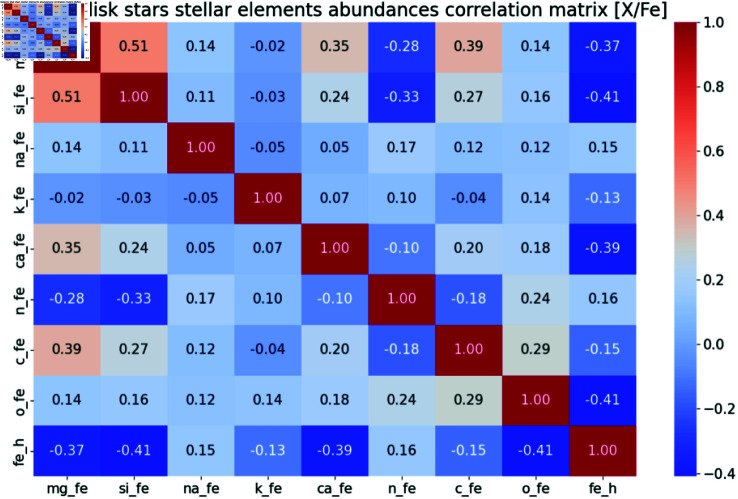
Example of correlation heatmap. Retrieved from Wikimedia Commons under the Creative Commons Attribution 4.0 International license.

After computing the quantitative statistical description of the dataset, we suggest to identify any potential correlation between variables, by employing common statistical tests and methods. For each pair of variables, we recommend to apply Pearson correlation coefficient (PCC), which captures any linear correlation between the two. The results of the Pearson correlation coefficient can then be employed to produce a correlation heatmap (Fig. 4).

A final useful step of exploratory data analysis is the visualization of the structure of the dataset. If a dataset has only two dimensions, a simple Cartesian scatterplot can be sufficient. With three dimensions, a heatmap can be informative. With four, or five dimensions, the additional variables in the Cartesian scatterplot can be represented through different shapes, different colors, and different sizes of the data points.

But what to do if there are more dimensions to represent? In these cases, that are common in biomedical sciences, one can apply dimensionality reduction methods, such as uniform manifold approximation and projection (UMAP) [31,32]. Applying UMAP to the original dataset and displaying its 2D representation can give insights about the structure of the datasets, highlighting special clusters of points that might correspond to particular groups of patients.

**Fig 5:**
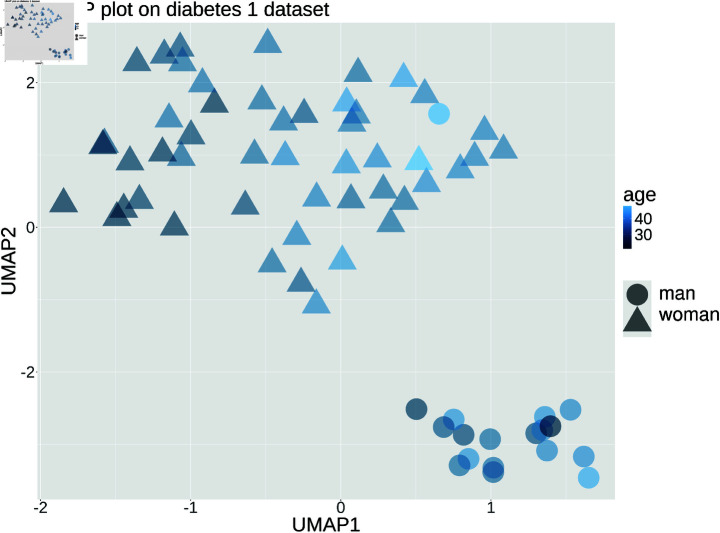
Example of UMAP representation of the diabetes type one dataset. We utilized the umap and the ggplot2 libraries on the [18] dataset of electronic medical records.

**2nd practical class: Exploratory data analysis.** We applied the exploratory data analysis (EDA) concepts seen during the previous class on the same dataset of the 1st practical lecture, containing EHRs of patients with type 1 diabetes. Initially, we installed the dlookr [30], tableone [33], and umap [34] R packages. Then, we loaded the dataset into memory and, as a first step, we generated some descriptive statistics of all the features involved, using the pastecs::stat.desc() and tableone::CreateTableOne() methods to derive additional quantitative descriptors. To analyze the distribution of the features, we generated histograms with the ggplot2 library. To identify the correlations between features, we computed three correlation matrices, utilized the *dlookr* package, with the Pearson correlation coefficient, the Kendall distance and the Spearman coefficient, respectively. We visually studied the corresponding correlation heatmaps like we did for the histograms, and interpreted their differences. Finally, to comprehend a bit of the hidden structure representation of the dataset, we applied the Uniform Manifold Approximation and Projection (UMAP) technique [35] for dimensionality reduction, using the *umap* R package. We performed a grid search optimization of the main hyper-parameters of UMAP, and then set the number of neighbors parameter to 20 and the minimum distance to 0.01. We represented the *age* and *sex* features as color and shape, respectively, of the points of a Cartesian scatterplot, along with the two dimensions resulting from the UMAP algorithm (Fig. 5). We additionally experimented some changes both in the number of neighbors and minimal distance parameters, and in the features represented as color and shape.

**5th lecture: Unsupervised machine learning (clustering).** Once the dataset has been efficiently preprocessed, cleaned, and prepared, and exploratory data analysis has been conducted, it is time to apply machine learning to infer new biomedical knowledge about the investigated disease or biological aspect. Unlike the previous steps, this machine learning phase can produce outcomes and results that, if confirmed, might lead to new medical or biological knowledge. This scientific progress could, optimistically, improve patients’ lives, which is the ultimate goal of biomedical research.

During the fifth lecture, we described and taught the main concepts of unsupervised machine learning for clustering. Cluster analysis, in fact, makes up a large portion of machine learning studies worldwide. In our class, we explained the main difference between supervised and unsupervised approaches, and explained two algorithms in detail: *k*-means [36] and hierarchical clustering [37].

On this topic, we leveraged this lesson on the material of Sherrie Wang at Stanford University [38]. We also briefly described some metrics for internal clustering assessment of convex-shaped clusters (Silhouette score [39], Davies-Bouldin index [40], Dunn index [41], Calinski-Harabasz index [42], and Gap statistic [43]) and the main metric for external clustering assessment (adjusted Rand index [44]).

**3rd practical lecture: Unsupervised machine learning (clustering).** During the third practical class, as a first step, we installed additional R libraries: factoextra [45], ggdendro [46], fpc [47], cluster [48], clusterSim [49], and parameters [50]. We standardized the features to have a mean of 0 and a standard deviation of 1. We applied the *k*-means algorithm [36] with the number of clusters set to 2. We visualized the projection of the two principal component analysis (PCA) of *k*-means results with the fviz_cluster() command, and then we assessed the performance of *k*-means by using already-mentioned five clustering internal metrics for convex clusters.

We repeated the *k*-means clustering analysis with the number of clusters set to 3, and compared the results with the previous ones to identify the best number of clusters.

In the second part of the lecture, we explored and studied a clustering technique from another clustering algorithm family: the hierarchical clustering method [51]. At first we selected the best linkage method among *average, single, complete,* and *Ward* [37]. The Ward linkage method generated the highest linkage score. We applied the hierarchical clustering with the hsclust() function, and visualized the resulting dendrogram. Using the cluster_analysis() command, we set the number of cluster to 2, and the same five clustering metrics that we used for the *k*-means results. Finally, we repeated the hierarchical clustering analysis with 3 cluster, and compared the results with the ones obtained with 2 clusters. The goal of this phase was to perform a minimal phase of optimization of the number of clusters for *k*-means, by also noticing that the five metrics employed can generate different outcomes.

**6th lecture: Supervised machine learning.** In this lecture, we outlined the main concepts of supervised machine learning, by also explaining the best practices of supervised computational intelligence in biomedical sciences [52–54]

We described key concepts such as the split between training set and test set, the difference between held-out validation and *k*-fold cross validation, the role of hyperparameters [55], the problems of overfitting [56], and the main metrics for binary classification result assessment (such as the Matthews correlation coefficient [57]) and for regression analysis result assessment (such as R^2^, the coefficient of determination [58]).

Also for this lesson we took advantage of some material of Sherrie Wang at Stanford University [38].

Eventually, we explained a popular algorithm of feature ranking based on supervised machine learning: recursive feature elimination (RFE) [59]. We also briefly described SHapley Additive exPlanations (SHAP) [60], another famous algorithm for the same scope, and we mentioned LASSO [61].

**4th practical class: Supervised machine learning.** In this practical class, we installed the following R packages: randomForest [62], metrics [63], and shapr [64]. We loaded the diabetes dataset and set the *insulin_regimen_binary* feature as the target variable. We randomly shuﬄed the rows of the dataset, and selected 80% of the rows (patients) for the training set and the remaining part for the test set [53]. We used the Random Forests [65] technique for binary classification and eventually computed the result metrics on the test set. We additionally computed the Matthews correlation coefficient (MCC) [57,66,67], and checked if the prediction were all of the same class: in this case it is not possible to compute the MCC across the folds training and inference phases. Using the *held-out* approach presented during the 6th lecture, we repeated the execution of the binary classification 1,000 times, by using randomly sampled data instances every time. We saved the MCC at each execution, then we computed its mean and standard deviation. We used the recursive feature elimination based on both the MCC and the precision metric to assess the most predictive variables. We implemented the splitting of the dataset to perform a 5-fold cross-validation procedure, and eventually computed the mean and standard deviation of the MCC. Finally, we applied the Shapley method to assess the features importance [60]. We used the shapr() command, and specified the expected prediction without any features. The actual Shapely values were computed with the kernelSHAP() function accounting for feature dependence. Finally, we plotted the explanation for two random observations.

After this last practical class, we asked to the students to repeat all these computational analyses on another dataset of EHRs of patients with diabetes type two [68].

**7th lecture: Result understanding and validation.** In the last lecture of our course, that corresponds to the last step of a biomedical data science project, we explained what to do to validate the results obtained in the previous steps.

Although pivotal, this phase is often overlooked by data scientists and researchers, who often wrongly believe that *results talk by themselves*. Here we explained to the students that validation can be internal or external.

Regarding internal validation, one can check if different computational methods produce similar results or check if different computational phases generate concordant results. The former case is quite common in computational projects. For example, regarding unsupervised clustering, a good idea is to apply different algorithms such as *k*-Means, DBSCAN (density-based spatial clustering of applications with noise), Hierarchical Clustering, BIRCH (balanced iterative reducing and clustering using hierarchies), and Mean-Shift, and to compare their results. If some specific clusters are identified by the majority of these methods, we can consider these clusters stable and reliable.

The same goes with supervised machine learning. One can apply Decision Trees, *k*-Nearest Neighbors, Random Forests, Naive Bayes, Support Vector Machines, Linear Regression and other methods on the same dataset and see if they obtain similar results. Moreover, comparing the results of feature ranking generated with supervised machine learning with the results of the same phase made through statistical tests (through Mann-Whitney *U* test [69], Kruskal-Wallis test [70], and chi-square test [71]) can be a good idea. For these biostatistics tests, during the lesson we reaffirmed the importance of using a 0.005 threshold for *p*-value significance, as suggested by Daniel J. Benjamin and colleagues [72], rather than using the traditional, too permissive 0.05 threshold. The 0.005 significance threshold, in fact, allows only the selection of strongly significant results. We also recommended to use the adjusted *p*-value rather than the nominal *p*-value, when present [73, Tip 5].

The feature rankings produced by the different methods can be compared through the Spearman *ρ* rank correlation coefficient or the Kendall *τ* distance [74].

Similarity between results can be found also by analyzing the outcomes of different computational phases. If the exploratory data analysis made through the Pearson correlation coefficient highlighted the association between the target variable and a specific feature, we expect to see this feature among the most relevant in the feature ranking machine learning phase outcome, too.

For external validation, we indicated three main approaches: one relying on external datasets, one relying on scientific literature, and one relying on external collaborators. After a data scientist completes their analysis on a dataset, that we can call *primary dataset*, she or he can look for an alternative, external *validation dataset* of the same disease, of the same data type, and possible having the same features. Of course, finding such dataset can be difficult, since there is a huge variety on data types and variables, but we suggest to give it a try anyway. To do so, we taught our students to use the already-mentioned search engines and repositories (Google Dataset Search, re3data.org, PhysioNet, Zenodo, Kaggle, UC Irvine ML Repository, Figshare, UK Biobank, and dbGaP, for example). If found, of course one can repeat their computational analysis on the validation dataset, and see if they obtain similar outcomes both on the primary and on the validation dataset.

Another form of validation involves the usage of the same database, but of versions refering to different times: you can perform some computational predictions on the oldest dataset and see if they were confirmed in the newest, most recent edition of the same dataset, retrospectively [75]. For example, one could make predictions of biomolecular annotations on the Gene Ontology (GO) database version of 2008 [76], and then see if these annotations were included in the Gene Ontology database version of 2023 [77].

External validation can be done also through two additional ways: by searching in the scientific literature and by involving a medical doctor. We recommend to look for articles the same contents of a study on search engines such as Google Scholar, Scopus, DBLP, PubMed, IEEE *Xplore*, and see how other scientists employed the same algorithms on similar datasets for similar goals. Regarding feature ranking, we suggest to look for articles confirming or rejecting the association between clinical variables and the analyzed disease. Finally, when the study is over and the results are clearly defined, we advice to go and talk with wet-lab biologist or a medical doctor who might be available to assess the outcomes of a data science study (Fig. 3). Their feedback would be invaluable.

## Conclusions

Data science has become a pivotal tool for biomedical research, and therefore teaching units and courses on this theme have spread in several universities worldwide. In this study, we reported and described the content of the course on biomedical data science that we gave to the master’s degree students of our university last year. As we explained, we reaffirmed the necessity to doubt and comprehend critically the results of any machine learning step, by avoiding the blind acceptance of the outcomes obtained. We believe that the topics described in this education article could be useful for anyone who needs to prepare a syllabus for a health data science course, anywhere around the world.

We are still awaiting the aggregated general feedback from the students regarding the short course, but our impressions during the classes were positive: considering the continuous interactions and the frequent questions they asked, we believe they appreciated the course contents and our teaching style.

Unfortunately, our short course was scheduled in the second semester of the second and final academic year of the Data Science master’s degree. This timing led to some students being *distracted* by other commitments, such as company internships and writing their master’s theses. As a result, some students rarely attended the lectures and opted to study the course material independently.

Regarding limitations, we have to admit that we had to neglect some particular biomedical data science tasks in our course, due to lack of time. We could not talk about the importance of batch correction [78] and broadly of noise removal [79], for example, which are important steps in bioinformatics and health informatics. In fact, we wanted to propose our contents as general as possible so that they could be applied to any data science study on any biomedical data type. Moreover, if additional lessons could be added to our course, it would be useful to include a class on ethical aspects of biomedical data science [80].
